# Lipoid Pneumonia: HRCT and MRI Spectrum, Diagnostic Pitfalls, and Imaging-Based Diagnostic Workflow

**DOI:** 10.3390/diagnostics16111693

**Published:** 2026-05-30

**Authors:** Miriam Adorna, Martina Contino, Alessandro Libra, Letizia Antonella Mauro, Davide Giuseppe Castiglione, Claudia Mattina, Claudio Mauceri, Claudia Crimi, Alberto Terminella, Giacomo Cusumano, Alessandra Gurrera, Pietro Valerio Foti, Gianluca Sambataro, Antonio Basile, Carlo Vancheri, Stefano Palmucci

**Affiliations:** 1 Training in Radiology Residency, Department of Medical Surgical Sciences and Advanced Technologies “GF Ingrasia”, University of Catania, 95123 Catania, Italy; miriamadorna@gmail.com (M.A.); martinacontino@gmail.com (M.C.); 2 Pulmonology Unit, University Hospital “Policlinico G. Rodolico-San Marco”, 95123 Catania, Italy; alessandrolibra@outlook.it (A.L.); claudia.crimi@unict.it (C.C.); vancheri@unict.it (C.V.); 3 Pulmonary Imaging and Advanced Radiological Techniques Unit (UOSD IPTRA), 95123 Catania, Italy; mauroletizia@tiscali.it; 4 Department of Medical Surgical Sciences and Advanced Technologies “GF Ingrassia”, University of Catania, 95123 Catania, Italy; davidegiuseppecastiglione@gmail.com (D.G.C.); c.mauceri@policlinico.unict.it (C.M.); pietrofoti@hotmail.com (P.V.F.); basile.antonello73@gmail.com (A.B.); 5 University Hospital Policlinico “G.Rodolico-San Marco”, 95123 Catania, Italy; claudiamattina0@gmail.com; 6 Department of Thoracic Surgery Unit, University Hospital Policlinico “G. Rodolico-San Marco”, 95125 Catania, Italy; a.terminella@ao-ve.it (A.T.); giacomo.cusumano@unict.it (G.C.); 7 Department of Pathology, University of Catania, 95123 Catania, Italy; alessandragurrera@libero.it; 8 Department of Medicine and Surgery, University of Enna “Kore”, 94100 Enna, Italy; dottorsambataro@gmail.com

**Keywords:** lipoid pneumonia, HRCT, fat attenuation, MRI, chemical shift imaging, diagnostic workflow, crazy paving, pulmonary consolidation, exogenous lipoid pneumonia, endogenous lipoid pneumonia

## Abstract

**Background/Objectives**: Lipoid pneumonia (LP) is a rare and frequently underdiagnosed pulmonary condition with a broad spectrum of radiological manifestations that can closely mimic infectious, inflammatory, and neoplastic lung diseases. Despite its clinical relevance, no standardized imaging-based diagnostic pathway exists. For this reason, this pictorial narrative review aims to provide a structured, imaging-centred synthesis of LP, to characterise the full spectrum of high-resolution CT (HRCT) and magnetic resonance imaging (MRI) findings, and to propose a pragmatic diagnostic workflow. **Methods**: A systematic literature search was performed in PubMed, MEDLINE, Embase, and the Cochrane Library from January 1950 to February 2025. Search terms combined “lipoid pneumonia” with imaging-related keywords including “HRCT,” “computed tomography,” “MRI,” and “fat attenuation.” After screening 891 deduplicated records, 60 studies were included in the narrative synthesis. Eight illustrative institutional cases with imaging–pathology correlation were additionally selected to demonstrate key imaging phenotypes. **Results**: HRCT is the cornerstone modality, demonstrating intralesional fat attenuation (typically −30 to −150 HU) in 40–80% of cases depending on series and disease chronicity. Additional patterns include ground-glass opacity, crazy paving, centrilobular nodules, and mass-like consolidation mimicking malignancy. Fat attenuation is absent in up to 60% of cases when inflammatory exudate or fibrosis masks lipid content. MRI, particularly chemical shift imaging, serves as a problem-solving adjunct in pseudotumoral or densitometrically equivocal presentations. A pragmatic diagnostic workflow is proposed, integrating HRCT findings, exposure history, fat-sensitive MRI in selected cases, BAL cytology, and histopathological confirmation when required. **Conclusions**: A pattern-based radiological approach, anchored on HRCT and integrated with clinical exposure history, BAL cytology, and selective use of fat-sensitive MRI, enables accurate diagnosis of LP in most cases and can prevent unnecessary invasive procedures including surgical resection performed under suspicion of malignancy.

## 1. Introduction

Lipoid pneumonia (LP) is an uncommon pulmonary condition characterised by the intra-alveolar accumulation of lipid material, leading to a variable inflammatory response and a broad spectrum of radiological manifestations. Although traditionally considered rare, its true prevalence is likely underestimated due to its nonspecific clinical presentation and its frequent radiological resemblance to infectious, inflammatory, and neoplastic lung diseases. The diagnosis is often delayed or incidental, and in some cases the disease is identified only after invasive diagnostic procedures performed for suspected malignancy. Available epidemiological data must be interpreted with awareness of their methodological limitations. Historical autopsy-based estimates—including early series reporting LP in up to 1–2.5% of unselected autopsies—reflect a context of widespread mineral oil use as a laxative and nasal lubricant, and are not applicable to contemporary clinical populations. More recent cohort data, including the Mayo Clinic series by Samhouri et al. (2021) [1], provide a more clinically grounded estimate but are subject to referral bias inherent in tertiary centre populations. A distinct epidemiological context emerged with the 2019 E-cigarette or vaping product use-associated lung injury (EVALI) outbreak in the United States, which brought LP to the attention of a broader medical community and demonstrated that the condition is not limited to elderly patients with aspiration risk factors. Together, these data sources suggest that LP is genuinely rare as a clinically recognised entity, but likely more prevalent than formally documented when subclinical and fat-negative forms are considered [1].

LP is broadly classified into exogenous and endogenous forms, depending on the origin of the lipid material. Exogenous lipoid pneumonia results from aspiration or inhalation of external fatty substances, whereas endogenous lipoid pneumonia develops secondary to airway obstruction or chronic parenchymal injury, leading to the release and accumulation of lipid-rich cellular breakdown products within the alveoli. Despite this etiological distinction, the two forms share overlapping pathological and imaging features, which may complicate diagnosis [2].

Imaging plays a pivotal role in recognizing this entity. HRCT is the cornerstone modality, allowing identification of characteristic parenchymal patterns and, in selected cases, direct detection of intrapulmonary fat through negative attenuation values. MRI may serve as a complementary tool in complex or mass-like presentations. This review aims to provide a structured, imaging-centred analysis of lipoid pneumonia, integrating pathophysiology, histopathology, and advanced imaging features, with particular emphasis on diagnostic pitfalls and complications. For this reason, a new diagnostic workflow is proposed in order to overcome the lack of standardized imaging-based diagnostic pathways described in the literature. Despite a growing body of literature, two clinically relevant gaps remain unaddressed: the variability of CT fat attenuation (reported in 40–80% of cases across series) is rarely contextualised in terms of methodological heterogeneity and no standardised imaging-based diagnostic pathway exists to guide clinicians from radiological suspicion to confirmed diagnosis. The emergence of the EVALI outbreak further reinforces the need for a contemporary, practically oriented imaging review addressing these gaps [3,4].

## 2. Materials and Methods

**Scheme 1:**
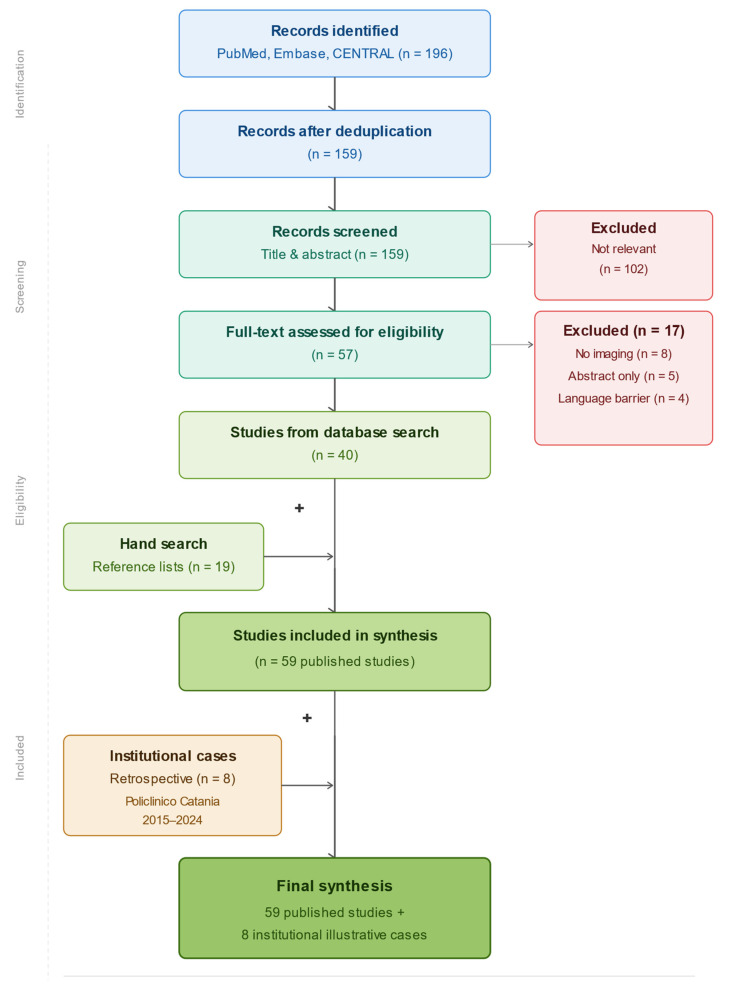
PRISMA-style flow diagram of the study selection process. The database search identified 196 records, of which 40 studies were eligible after screening and full-text assessment; 19 additional studies were retrieved by hand search. The final synthesis included 59 published studies and 8 retrospective institutional illustrative cases from Policlinico Catania, 2015–2024.

This narrative pictorial review was conducted according to the general principles of the PRISMA 2020 statement, adapted to the narrative design of the study. A comprehensive literature search was performed in PubMed/MEDLINE, Embase, and the Cochrane Central Register of Controlled Trials, covering the period from January 1950 to 3 February 2026 (Scheme 1) (Supplementary Material S1).

The following Boolean search strategy was applied in PubMed/MEDLINE:

(“lipoid pneumonia” [MeSH Terms] OR “lipoid pneumonia” [Title/Abstract] OR “lipid pneumonia” [Title/Abstract] OR “exogenous lipoid pneumonia” [Title/Abstract] OR “endogenous lipoid pneumonia” [Title/Abstract] OR “cholesterol pneumonia” [Title/Abstract]) AND (“computed tomography” [Title/Abstract] OR “CT” [Title/Abstract] OR “HRCT” [Title/Abstract] OR “high-resolution CT” [Title/Abstract] OR “magnetic resonance imaging” [Title/Abstract] OR “MRI” [Title/Abstract] OR “fat attenuation” [Title/Abstract] OR “chemical shift” [Title/Abstract] OR “imaging” [Title/Abstract] OR “radiology” [Title/Abstract] OR “radiological” [Title/Abstract]).

Equivalent strategies using Emtree terms and free text were applied in Embase (https://www.embase.com, accessed 3 February 2026), while free text terms were used in CENTRAL. Additional studies were identified through manual screening of reference lists from eligible articles and relevant reviews. No language restriction was applied at the search stage.

All retrieved records were exported to a reference manager and duplicates were removed. Two authors independently screened titles and abstracts, with disagreements resolved by consensus. Full texts were assessed when potentially eligible. Studies were included if they reported confirmed exogenous or endogenous lipoid pneumonia, provided original CT, HRCT, or MRI imaging data, and were published as full text articles in peer-reviewed journals. Diagnostic confirmation required histopathology, BAL cytology with positive Oil Red O staining, or characteristic imaging findings combined with a compatible clinical exposure history. Studies without radiological data, abstract only reports, animal or in vitro studies, and incidental autopsy only diagnoses were excluded.

The initial search retrieved 196 records. After removal of 37 duplicates, 159 unique records were screened and 102 were excluded as clearly non relevant. Fifty-seven full text articles were assessed for eligibility; 17 were excluded because of absent original imaging data(*n* = 8), abstract only publication (*n* = 5), or language barrier without English abstract, (*n* = 4). Overall, 40 studies were identified through database searching and 19 additional studies through hand search, resulting in 59 published studies included in the narrative synthesis. Given the predominance of case reports and small case series, formal quality assessment with standardised tools was not performed. Instead, studies were prioritised according to imaging quality, sample size, and availability of radiological, pathological, or BAL correlation. In parallel, a retrospective review of the radiological archive of the University Hospital “Policlinico G. Rodolico, San Marco,” University of Catania, Italy, was performed to identify institutional cases of confirmed lipoid pneumonia between January 2015 and December 2024. Final diagnosis was established during a multidisciplinary meeting involving thoracic radiologists, pulmonologists, thoracic surgeons, and pathologists. A total of eight institutional cases were selected by two experienced thoracic radiologists by consensus. Selection was based on the ability to illustrate relevant imaging phenotypes or diagnostic pitfalls, the availability of high-quality digital imaging suitable for publication, and the presence of diagnostic confirmation, preferably histopathological or cytological. The retrospective use of anonymised and de-identified imaging data was conducted in accordance with the Declaration of Helsinki. Because of the retrospective design, complete anonymisation, and absence of experimental intervention, formal ethics committee approval was waived according to institutional policy.

## 3. Discussion

### 3.1. Epidemiology

Lipoid pneumonia (LP) is a rare and likely underdiagnosed condition, and precise incidence and prevalence data are lacking. Contemporary population-based estimates are unavailable, and no national surveillance systems currently track the disease. On the other hand, a retrospective study from the Mayo Clinic identified 34 cases of exogenous LP over a 22-year period (1998–2020), underscoring its rarity even in tertiary referral centres [5]. Moreover, no reliable geographic prevalence differences have been established [6]. Historical autopsy series reported LP in approximately 1.0–2.5% of adults and up to 8.8% of children, while an older study (1951) found evidence of LP in 14.6% of 389 chronically ill patients [1].

### 3.2. Definition and Classification

Lipoid pneumonia (LP) is a rare diffuse pulmonary disease, where there is an accumulation of fatlike compounds in the distal airways and alveoli, causing an inflammatory reaction [7].

There are two types of LP, the endogenous form and the exogenous form.

Endogenous LP, also known as “cholesterol” or “golden” pneumonia, was first described by McDonals et al. in 1949 [8]. The condition is caused when the lipids that form the lung tissue are released due to the rupture of the alveolar cell walls distal to an obstructive lesion, due to a suppurative process, or due to lipid storage diseases, disorders of lipid metabolism, or immunologic and rheumatologic disorders that cause chronic inflammation (juvenile rheumatoid arthritis, Hodgkin’s lymphoma, primary sclerosing cholangitis, pulmonary alveolar proteinosis, Wegener’s granulomatosis, etc.) [9].

Exogenous LP was first described by Laughlen in patients with a history of chronic laxative and nasal drop use [10] and can be divided in acute and chronic. The acute form occurs when an individual accidentally inhales a large quantity of lipid material in a short period of time, while the chronic form is due to a recurrent inhalation of lipidic compounds [11].

The risk factors for exogenous LP are multiple [12].

- Anatomic defects (cleft palate, anatomical pharyngeal and oesophageal abnormality).
- Neuromuscular diseases (functional pharyngeal and oesophageal abnormality).
- Occupation related.
- Drugs (laxatives, lip balm, oily nasal drops) [13].

Exogenous LP is most commonly associated with aspiration or inhalation of mineral oils (e.g., oil-based laxatives, nasal drops), food oils, or lipid-containing vaping products, particularly THC oils. The disease affects all age groups but shows a predilection for the extremes of life, especially children and elderly patients with predisposing factors such as dysphagia or gastroesophageal reflux [14]. In the Mayo cohort, the median age was 71 years (IQR 59–76), with no significant sex predominance; approximately half of patients were asymptomatic at presentation. In contrast, during the 2019 EVALI outbreak, many cases of acute lipid-related lung injury occurred in young adults (18–35 years) using THC-containing e-cigarettes [15,16]. Overall, sex distribution appears balanced, although available series are too small to confirm meaningful differences. Mortality rates are not well defined, but severe acute presentations, including ARDS, may require intensive care and systemic corticosteroid therapy [17].

### 3.3. Pathophysiology and Histopathology

The pathophysiological mechanisms of lipoid pneumonia differ according to its aetiology but ultimately converge on similar inflammatory pathways.

In exogenous lipoid pneumonia, aspirated lipid droplets reach the distal airways and are phagocytosed by alveolar macrophages. However, mineral oils and other lipid substances are poorly metabolized by these cells. The persistence of lipid-laden macrophages leads to cellular degeneration, release of intracellular lipid content, and a chronic foreign-body inflammatory reaction. Over time, this process may evolve into granulomatous inflammation and progressive interstitial fibrosis [18].

These pathological events directly underlie the imaging findings observed on HRCT: the accumulation of lipid-laden macrophages within relatively intact airspaces produces areas of consolidation with negative attenuation values (typically −30 to −150 HU), reflecting the preponderance of lipid material. When inflammatory exudate or interstitial oedema is superimposed, a ground-glass opacity or crazy paving pattern results—the latter arising from inflammatory interlobular septal thickening overlying a background of ground-glass change. As the foreign-body giant cell reaction intensifies and fibroblastic repair is initiated, progressive fibrosis produces HRCT features of reticulation, traction bronchiectasis, and architectural distortion [19,20]. In pseudotumoral LP, the mass-like consolidation reflects a densely packed collection of foam cells and fibrous stroma, producing a solid lesion indistinguishable morphologically from primary lung carcinoma—a scenario in which chemical shift MRI has particular added diagnostic value [21].

In endogenous lipoid pneumonia, airway obstruction or chronic parenchymal injury results in the degeneration of pneumocytes and macrophages, releasing cholesterol and lipid-rich membrane components into alveolar spaces. Accumulation of cholesterol clefts and foamy macrophages perpetuates inflammation and may contribute to fibrotic remodelling [22].

**Figure 1:**
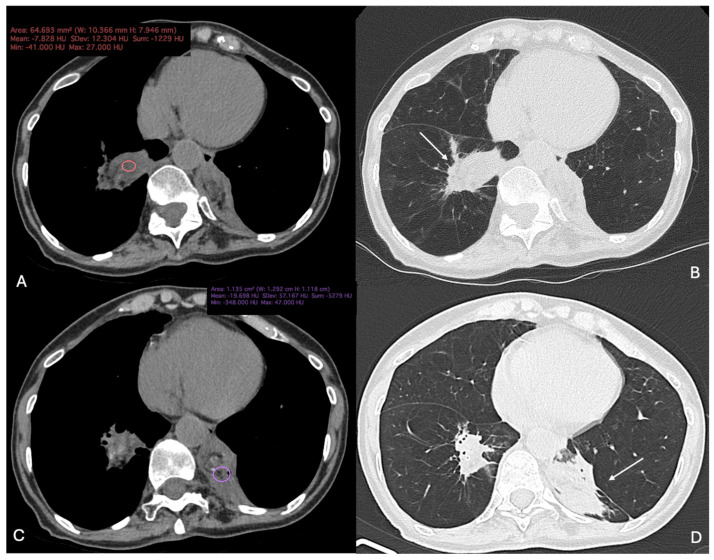
(**A**,**B**) Axial non-contrast HRCT images in mediastinal (**A**) and lung (**B**) windows in a woman with chronic aspiration of nasal allergy spray depict a mass-like consolidation without air bronchogram in the lateral and posterior segments of the right lower lobe, characterized by irregular spiculated margins (arrow in (**B**)). The consolidation shows negative attenuation values (mean −8 HU), indicating intralesional fat (red circle in (**A**)). Despite the spiculated morphology, lung biopsy confirmed the diagnosis of lipoid pneumonia. (**C**,**D**) Axial non-contrast HRCT images in mediastinal (**C**) and lung (**D**) windows in a patient with chronic aspiration of chemical lubricant reveal a mass-like consolidation involving the superior and basal segments of the left lower lobe (arrow in (**D**)). Mean attenuation measures −20 HU, consistent with lipid content within the lesion (purple circle in (**C**)).

Histologically, both forms are characterized by the presence of lipid-laden macrophages filling the alveoli, often accompanied by multinucleated giant cells, cholesterol clefts, and varying degrees of interstitial fibrosis. Special stains such as Oil Red O and Sudan Black can confirm intracellular lipid content in appropriately preserved specimens [23]. Bronchoalveolar lavage demonstrating abundant lipid-laden macrophages often provides the key diagnostic confirmation, although the finding must be interpreted cautiously in the presence of obstructive lesions (Figure 1) [24,25].

### 3.4. Imaging

Imaging findings in lipoid pneumonia are diverse and reflect the stage and chronicity of the disease. Conventional chest radiography typically shows nonspecific opacities, including focal or diffuse consolidation and interstitial infiltrates. HRCT provides a more detailed and informative assessment.

#### 3.4.1. HRCT Protocol

HRCT examinations were performed using a multidetector CT scanner with thin-section volumetric acquisition. Technical parameters included slice collimation below 1.5 mm, typically ranging between 0.5 and 1.25 mm, with a reconstruction interval between 0.5 and 1.5 mm. Images were reconstructed using a high-spatial-frequency (sharp) kernel to optimize assessment of parenchymal structures. Image interpretation was performed using dedicated lung and mediastinal window settings. Mediastinal structures, including lymph nodes, esophageal caliber, and vascular measurements such as the pulmonary artery-to-aorta ratio, were assessed using standard mediastinal window settings.

#### 3.4.2. MRI Protocol

MRI is not a first-line modality for LP but serves as a problem-solving adjunct in selected clinical scenarios: pseudotumoral or mass-like presentations, densitometrically equivocal consolidations, and cases where avoiding invasive biopsy is a priority. When performed, examinations were acquired on a 1.5 T or 3.0 T system using a phased-array thoracic coil. The key sequences for LP characterisation are (1) T1-weighted imaging, where lipid-containing consolidations may show relatively increased signal intensity compared with skeletal muscle; (2) fat-suppressed T1-weighted sequences, where signal reduction confirms intralesional lipid; and (3) chemical shift gradient-echo imaging (in-phase and out-of-phase acquisitions), where signal drop on opposed-phase images indicates microscopic fat components—particularly useful when CT densitometry is equivocal. T2-weighted, diffusion-weighted (DWI), and contrast-enhanced sequences may further characterise lesion composition and vascularity but are not diagnostic for LP in isolation.

A particular focus is placed on fat-suppressed sequences and chemical shift imaging, given their key role in detecting intralesional fat.

Fat-suppressed T1-weighted sequences were included to confirm the presence of lipid content. In lesions containing macroscopic or microscopic fat, signal attenuation on fat-suppressed images compared with non–fat-suppressed sequences supported intralesional lipid composition.

When available, in-phase and out-of-phase gradient-echo sequences were performed to detect microscopic fat through signal drop on out-of-phase images. This approach was particularly relevant in cases where CT attenuation measurements were inconclusive due to inflammatory superimposition [26].

The standard protocol included axial T1-weighted sequences, axial and coronal T2-weighted sequences with and without fat suppression, diffusion-weighted imaging (DWI), and, when clinically indicated, contrast-enhanced T1-weighted three-dimensional gradient-echo sequences.

### 3.5. Imaging Features

#### 3.5.1. HRCT Features

HRCT represents the imaging modality of choice in the evaluation of lipoid pneumonia. The polymorphous spectrum of findings reflects the stage of the disease, the type of lipid exposure, and the relative contribution of intra-alveolar fat, inflammation, and fibrosis.

In exogenous LP, the dominant pattern is alveolar consolidation, typically segmental or patchy, frequently bilateral and gravity-dependent, with a predilection for the posterior segments of the lower lobes and the right middle lobe [27]. Consolidations are usually non-lobar, often demonstrate air bronchograms, and may show heterogeneous internal attenuation. The key diagnostic feature in exogenous lipoid pneumonia is the presence of intralesional fat attenuation, with measured values ranging from approximately −30 to −150 Hounsfield units. Regarding this, accurate placement of regions of interest within the most homogeneous portion of the consolidation is critical to avoid partial volume effects and misinterpretation (Figure 1).

**Figure 2:**
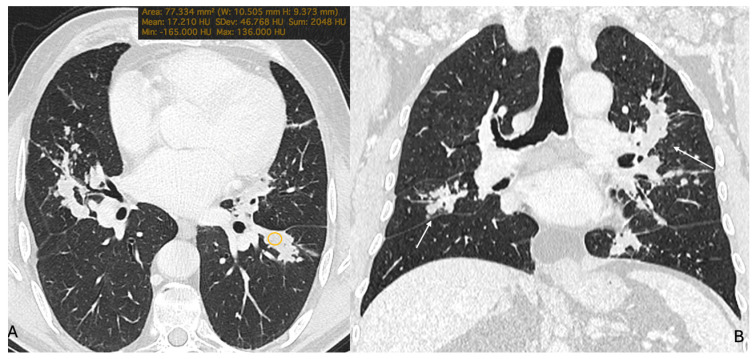
Axial lung-window CT (**A**) and coronal reformatted image (**B**) demonstrate a predominantly peribronchovascular nodular pattern, with clustered centrilobular/peribronchial nodules and branching opacities tracking along the bronchovascular bundles, most conspicuous in the middle lobe and lingula. Although this appearance can mimic infectious bronchiolitis or other inflammatory airway-centred processes, even for the the avarage positive density (Region of Interest rapresented by yellow circle in (**A**)), diagnosis is a biopsy-proven LP.

However, intralesional fat attenuation within a pulmonary consolidation on CT is not consistently demonstrable in all patients. For this reason, multiple studies show that fat may be masked when consolidation contains a mixture of lipid with inflammatory exudate or fibrosis, resulting in attenuation that is low but not, strictly speaking, “fat,” or even soft-tissue density [28] (Figure 2).

For example, in the largest modern adult cohort (34 adults evaluated at Mayo Clinic in 2021), fatty attenuation on CT was identifiable in only 41% of patients, underscoring that its absence does not exclude the diagnosis [1]. In contrast, several smaller HRCT-focused series (often enriched for classic oil aspiration and HRCT review) report fat-density (negative HU) consolidation in roughly 70–80% of patients, such as 80% in an Italian radiology cohort (10 cases) [29] and 70.6% in a paediatric mineral-oil series (17 cases) [30,31]. Taken together, published data suggest that visible fat-density consolidation is present in a substantial—but variable—proportion of patients (approximately 40–80%), likely influenced by disease chronicity and admixture of inflammatory exudate/fibrosis that can raise attenuation. Table 1 indicates the primary studies and large cohorts that provide frequencies of fat attenuation on HRCT.

**Figure 3:**
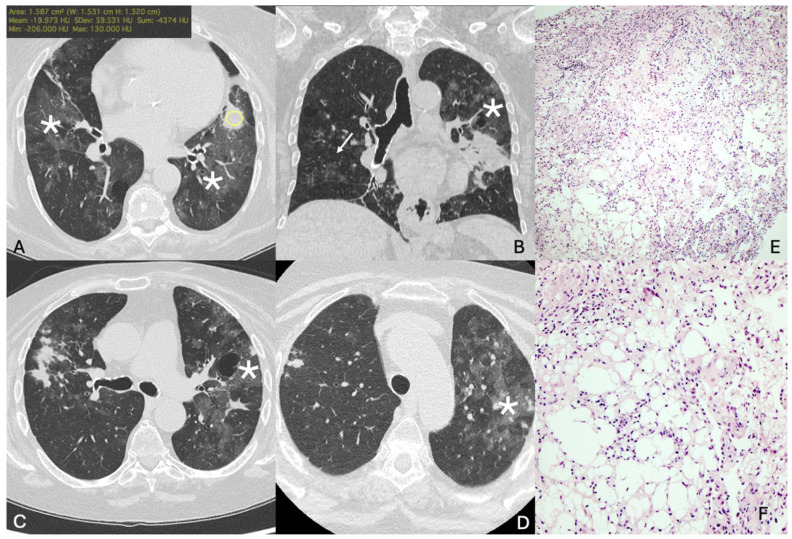
(**A**–**D**) Axial (**A**,**C**,**D**) and coronal (**B**) CT images demonstrate extensive bilateral ground-glass opacities (asterisks and arrow), representing the predominant imaging pattern in this case. Within the affected parenchyma, focal areas of consolidation with negative attenuation values (yellow circle (**A**)) are visible, consistent with intralesional lipid content. Additionally, multiple nodular consolidations with a peribronchovascular distribution are observed, particularly in the perihilar regions and along the bronchovascular bundles. This combination of ground-glass opacities, fat-density consolidations, and peribronchovascular nodular opacities represents a characteristic imaging spectrum of lipoid pneumonia. Histologic examination of the transbronchial lung biopsy confirms the nature of lipoid pneumonia. (**E**) Low-power view showing lung parenchyma involved by chronic inflammatory infiltrates and numerous optically empty vacuoles. (**F**) Higher-power view demonstrating variably sized lipid vacuoles, representing dissolved lipid material during histologic processing, surrounded by histiocytes and chronic inflammatory cells.

Ground-glass opacities are common and may appear diffuse or patchy, frequently coexisting with consolidations (Figure 3). They represent partial alveolar filling and interstitial inflammation and are often distributed in dependent regions. In acute presentations, ground-glass opacity may predominate before progression to denser consolidation [33].

**Figure 4:**
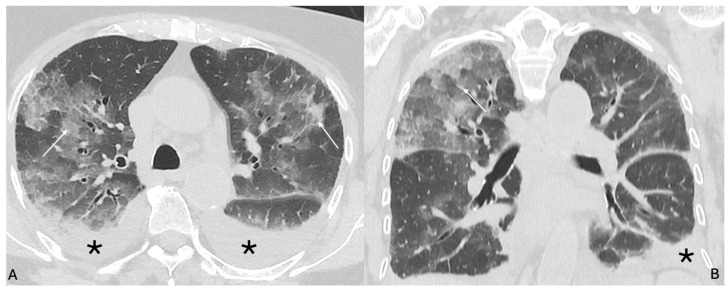
Axial non-contrast chest CT (**A**) and coronal reformatted image (**B**) demonstrate bilateral, predominantly upper-lobe ground-glass opacities with a perihilar distribution. At the level of the apical segments—particularly in the right upper lobe (white arrows)—a superimposed crazy paving pattern is evident, characterized by ground-glass attenuation associated with interlobular septal thickening and fine intralobular reticulation. Bilateral pleural effusions are present (asterisks), more pronounced on the left, with associated dependent posterior basal atelectatic consolidation. In the appropriate clinical context, these findings are suggestive of lipoid pneumonia; however, differential diagnoses including pulmonary oedema, infectious pneumonia, and other interstitial processes should also be considered.

Superimposed interlobular and intralobular septal thickening produces a crazy paving pattern, reported in a substantial proportion of cases. This pattern reflects the combined effect of intra-alveolar lipid material and interstitial inflammatory reaction (Figure 4) [34].

Although nonspecific, its association with areas of negative attenuation markedly increases diagnostic confidence. Distribution is often dependent, with a predilection for the posterior segments of the lower lobes in cases related to aspiration. However, unilateral or focal involvement may occur, particularly in chronic forms [32]. Centrilobular nodules are frequently observed, particularly in exogenous forms. These appear as poorly defined nodular opacities centred within secondary pulmonary lobules and may cluster or partially coalesce, occasionally mimicking a tree-in-bud configuration without true endobronchial impaction [35]. These correspond histologically to lipid-laden macrophage accumulation and peribronchiolar inflammation. In chronic disease, focal or dominant mass-like lesions may develop, characterized by irregular margins and heterogeneous density. Careful evaluation often reveals focal areas of negative attenuation within these pseudotumoral consolidations, representing retained lipid material. In some cases, however, intense inflammatory reaction may elevate attenuation values, rendering differentiation from primary lung carcinoma challenging [36].

**Figure 5:**
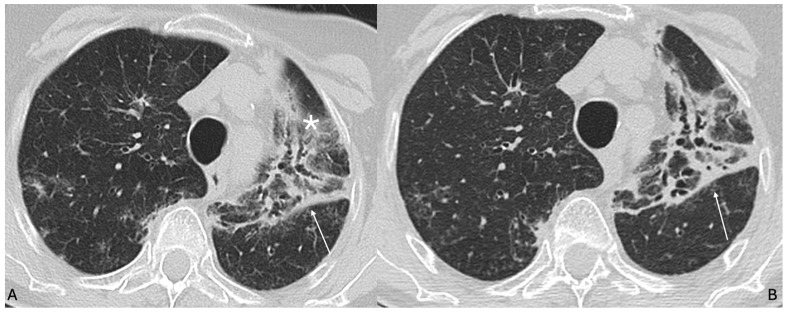
(**A**) Baseline HRCT shows extensive ground-glass opacities (asterisk), associated with heterogeneous consolidations and areas of mixed attenuation (arrow), consistent with an active inflammatory phase. (**B**) One-year follow-up HRCT demonstrates partial reduction of ground-glass opacities but progressive fibrotic changes (arrow), including reticular opacities, interlobular septal thickening, architectural distortion, and early traction bronchiectasis. These sequential images illustrate a potential disease evolution in lipoid pneumonia, with replacement of potentially reversible ground-glass abnormalities by irreversible fibrotic remodelling and parenchymal distortion, even after cessation of lipid exposure.

With disease progression, chronic lipoid pneumonia may lead to structural remodelling manifested by traction bronchiectasis, architectural distortion, subsegmental volume loss, and irregular reticulation within previously involved regions. Honeycombing is uncommon. The fibrotic changes tend to follow the distribution of prior consolidations rather than exhibiting the classic basal-subpleural predominance seen in idiopathic pulmonary fibrosis. Subpleural sparing is not a characteristic feature, and peripheral involvement is frequent [37] (Figure 5).

**Figure 6:**
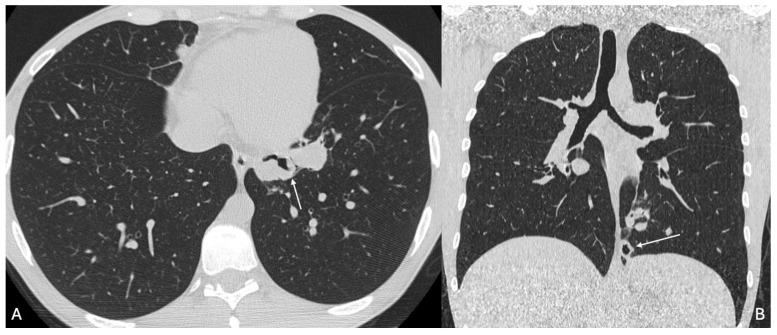
Axial lung-window CT (**A**) and coronal reformatted image (**B**) show focal cystic air-filled spaces consistent with pneumatoceles within/adjacent to a peribronchial area of parenchymal involvement in the lower lobe (white arrows). These thin-walled cavitary changes likely reflect airway-centred inflammatory injury with a check-valve mechanism and represent a potential complication/evolution in lipoid pneumonia in the appropriate clinical and histopathologic setting.

Airway abnormalities may include bronchial wall thickening and mild bronchiolar dilatation, particularly in cases of repeated aspiration. Expiratory imaging may demonstrate areas of air trapping secondary to small airway involvement. In severe acute hydrocarbon aspiration, HRCT may show extensive bilateral ground-glass opacities and consolidations, occasionally accompanied by pleural effusion. Rarely, cavitation or pneumatoceles have been reported in fulminant cases [38] (Figure 6).

Endogenous lipoid pneumonia generally presents as consolidation distal to an obstructive lesion [39]. In such cases, attenuation values may not demonstrate clear fat density, and the imaging appearance may be indistinguishable from post-obstructive pneumonia or tumour-related consolidation. Identification of an underlying bronchial obstruction is therefore essential. Associated findings may include segmental atelectasis, mucus plugging, or bronchial narrowing [40].

Less common CT manifestations include cavitation, pneumatoceles, pneumothorax, and pneumomediastinum, particularly in severe acute cases. Chronic cases may demonstrate bronchiectasis and residual fibrotic bands, reflecting long-standing inflammatory remodelling [41].

#### 3.5.2. MRI Features

MRI is not routinely considered as a primary diagnostic modality for LP. In standard clinical workflows HRCT remains the cornerstone because it provides superior spatial resolution for parenchymal characterization and, critically, enables objective confirmation of lipid content through attenuation measurements within the abnormality. Early thoracic MRI literature already framed MRI as potentially suggestive, but diagnostically limited: T1 hyperintensity may be encountered in lipid-containing consolidations even though it might be confusing as in case of proteinaceous material, subacute haemorrhage, high cellularity, or paramagnetic effects. By contrast, CT-based identification of intralesional negative attenuation values offers a more direct, quantifiable signature of fat within the lesion [13].

The clinical utility of MRI therefore lies in a narrow “problem-solving” niche, primarily when the dominant question is not diffuse ILD phenotyping but the characterization of a focal opacity with oncologic implications, namely the differentiation between pseudotumoral lipoid pneumonia and lung malignancy. This is most relevant in mass-like or nodular consolidations that prompt biopsy-driven pathways and, in reported adult cohorts, have occasionally led to surgical resections performed under suspicion of cancer, illustrating a scenario in which an additional non-invasive modality could be clinically valuable when available. A second context is equivocal or unreliable CT densitometry, for example in markedly heterogeneous consolidations, small foci prone to partial-volume averaging, motion-limited acquisitions, or lesions in which inflammatory exudation and fibrosis dominate and attenuate the macroscopic fat signal, thereby weakening the discriminatory power of HU measurements [1,42].

When applied in these selected settings, MRI can increase diagnostic confidence by leveraging fat-sensitive techniques that interrogate lipids at a microstructural level. These findings are best interpreted as complementary rather than definitive: MRI does not supplant HRCT, but may refine the differential diagnosis, support a lipid-related aetiology in pseudotumoral or densitometrically ambiguous cases, and help tailor the diagnostic trajectory, potentially mitigating unnecessary invasive procedures when imaging and clinical exposure history are concordant. On T1-weighted imaging, areas of consolidation containing lipid material may demonstrate relatively increased signal intensity when compared with skeletal muscle. However, signal intensity is typically lower than subcutaneous fat and may vary depending on the proportion of inflammatory exudate and fibrotic tissue. The presence of intralesional fat can be further evaluated using fat-suppressed T1-weighted sequences. Signal reduction on fat-suppressed images supports lipid content within the lesion.

**Figure 7:**
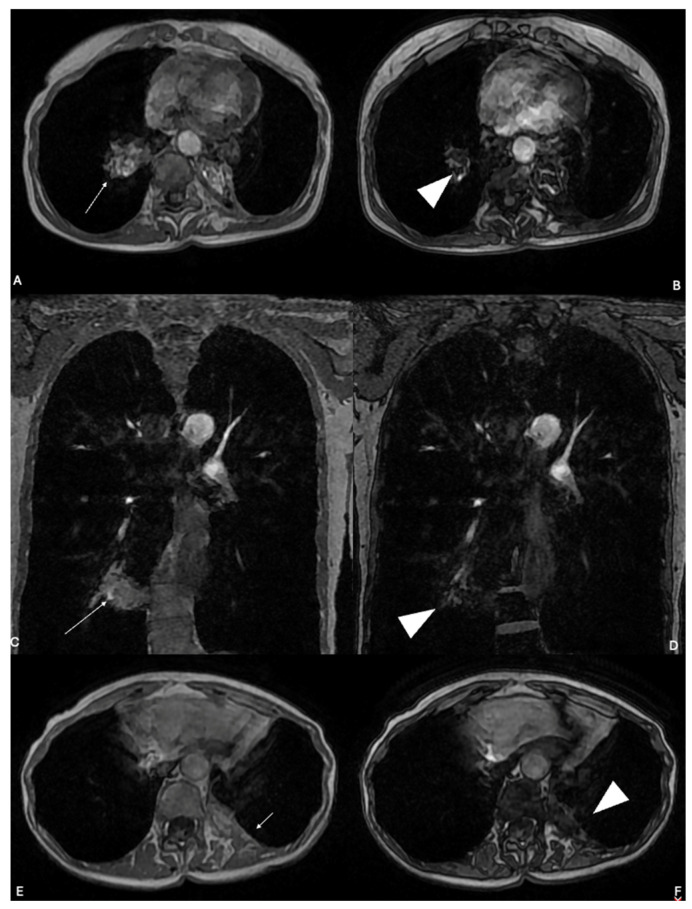
Chemical-shift chest MRI with paired in-phase and opposed-phase (out-of-phase) gradient-echo images: axial in-phase (**A**) and corresponding axial opposed-phase (**B**); coronal in-phase (**C**) and corresponding coronal opposed-phase (**D**); additional axial in-phase (**E**) and corresponding axial opposed-phase (**F**). Focal pulmonary lesions (arrows) show a conspicuous signal drop on opposed-phase images compared with in-phase images, consistent with intracellular lipid. Arrowheads indicate the corresponding opposed-phase signal loss/chemical-shift (“India ink”) effect at lesion margins, supporting fat-containing inflammatory deposits in the appropriate clinical context of lipoid pneumonia.

Chemical shift imaging may demonstrate signal drop on out-of-phase sequences, suggesting microscopic fat components. This technique is particularly useful when CT attenuation measurements are equivocal or when inflammatory components obscure clear fat-density values [26] (Figure 7).

**Figure 8:**
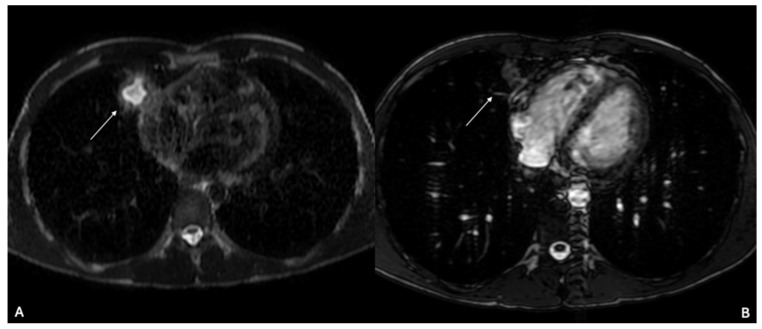
Axial T2-weighted fast spin-echo (FSE) image (**A**) shows a complex pulmonary lesion in the left lung (arrow) with marked T2 hyperintensity, consistent with a necrotic–liquefactive component complicating underlying lipoid pneumonia. Axial balanced steady-state free precession sequence (FIESTA) (**B**) (arrow) better delineates the lesion contours and its relationships with adjacent mediastinal structures, aiding pre-procedural/surgical planning and assessment for mediastinal contact or involvement.

On T2-weighted imaging, signal characteristics are heterogeneous. Areas of active inflammation and alveolar filling typically demonstrate intermediate to high signal intensity, whereas more fibrotic components may appear relatively hypointense. The coexistence of lesions at different stages of evolution may produce a mixed signal pattern within the same lobe (Figure 8).

Diffusion-weighted imaging may show high signal intensity on high b-value images in regions of dense inflammatory infiltration. However, restricted diffusion is not specific for lipoid pneumonia and may also be observed in neoplastic processes. Apparent diffusion coefficient values must therefore be interpreted in conjunction with morphological findings and clinical context. 

After administration of gadolinium-based contrast agents, lesions may demonstrate peripheral or heterogeneous enhancement, reflecting inflammatory hyperaemia rather than neoplastic angiogenesis. Enhancement patterns alone are insufficient for differentiation from malignancy and should be correlated with non-contrast sequences and CT attenuation characteristics [43].

### 3.6. Diagnostic Workflow

**Figure 9:**
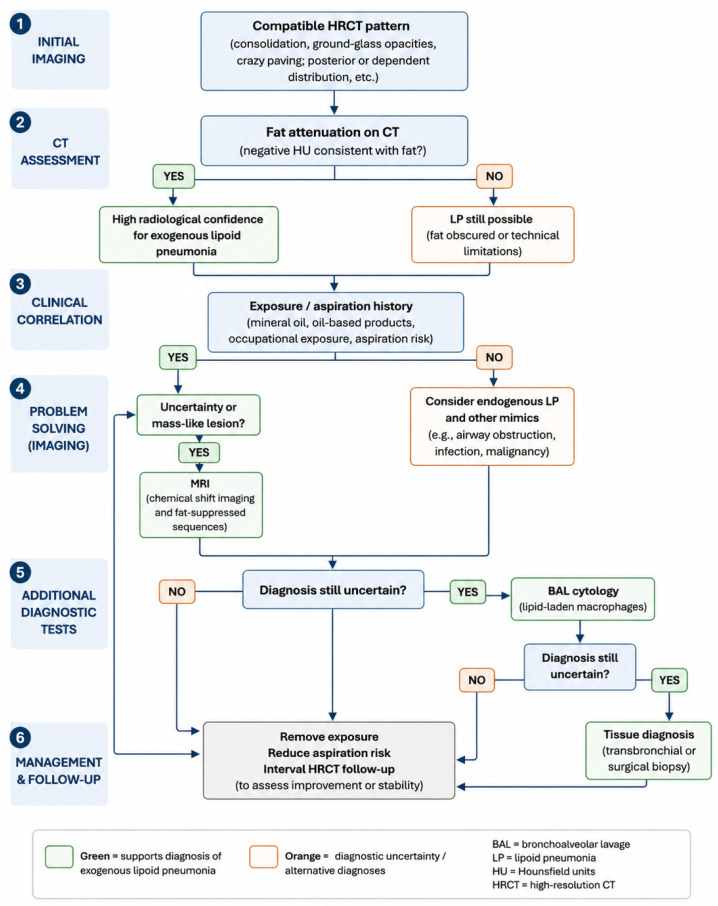
Diagnostic workflow to follow in order to diagnose lipid pneumonia.

To improve clinical applicability, the proposed diagnostic workflow (Figure 9) is structured around key decision points, reflecting real-world diagnostic reasoning rather than a purely descriptive approach.

The process begins with HRCT, which establishes the imaging pattern and identifies patients with a compatible phenotype (e.g., consolidation, ground-glass opacities, or crazy paving with posterior or dependent distribution).

The first critical decision point is CT densitometry. The demonstration of negative attenuation values consistent with fat strongly supports exogenous lipoid pneumonia and, when combined with a compatible clinical context, may allow a non-invasive working diagnosis. However, the absence of measurable fat does not exclude the disease, due to admixture with inflammatory or fibrotic components and technical limitations [19].

The next step is a focused exposure assessment, including mineral oil ingestion, oil-based products, occupational exposure, and risk factors for aspiration. When a plausible exogenous source is identified, diagnostic confidence increases.

In cases with equivocal densitometry, atypical imaging, or focal/mass-like presentation, MRI with chemical shift imaging and fat-suppressed sequences may serve as a problem-solving tool to support the presence of intralesional lipid.

If diagnostic uncertainty persists, bronchoalveolar lavage (BAL) may provide supportive evidence through the identification of lipid-laden macrophages.

However, this finding is not specific, as lipid-laden macrophages may also be observed in other conditions, including aspiration of gastric contents, chronic inflammation, or airway obstruction. In addition, sampling variability and the focal nature of disease may result in false-negative or inconclusive BAL findings, particularly in localized or mass-like presentations [44]. Therefore, BAL should be considered a context-dependent adjunct, rather than a standalone diagnostic test.

Histopathological confirmation remains the reference standard, particularly when malignancy, infection, or other mimics cannot be confidently excluded [45]. Nonetheless, biopsy is not without limitations. Sampling error may occur in heterogeneous lesions, and invasive procedures carry procedural risks, which must be weighed against the expected diagnostic yield [46].

In the absence of an identifiable exogenous source, the workflow shifts toward endogenous lipoid pneumonia and alternative diagnoses, including airway obstruction and post-obstructive changes, which should be actively investigated.

Finally, once the diagnosis is established or considered highly probable, management-oriented steps—including removal of exposure, reduction of aspiration risk, and interval HRCT follow-up—are essential both for treatment and for indirect diagnostic confirmation.

As illustrated in Figure 9, this algorithm emphasizes a stepwise escalation from non-invasive imaging to invasive confirmation, balancing diagnostic confidence with procedural risk.

### 3.7. Complications of Lipoid Pneumonia

Lipoid pneumonia may follow a benign and self-limited course after cessation of exposure; however, in a subset of patients, the inflammatory process may persist or progress, leading to structural, infectious, and diagnostic complications. The type and severity of complications depend on the amount and chronicity of lipid exposure, host immune response, and the presence of underlying pulmonary diseas (Table 2).

#### 3.7.1. Structural Complication (Fibrosis and Bronchiectasis)

One of the most relevant long-term complications of lipoid pneumonia is the development of interstitial fibrosis. Persistent intra-alveolar lipid accumulation triggers a chronic inflammatory reaction characterized by macrophage infiltration, foreign-body giant cell response, and eventual fibroblast activation. Over time, this may result in remodelling of the secondary pulmonary lobules.

On HRCT, fibrotic progression manifests as reticulation, architectural distortion, traction bronchiectasis, and localized volume loss. In chronic exogenous forms, fibrotic changes are often segmental or lobar, predominantly involving dependent regions. Unlike idiopathic pulmonary fibrosis, the distribution is typically focal and correlates with prior areas of lipid consolidation.

This fibrotic evolution may explain the persistence of radiologic abnormalities even after removal of the offending agent. In advanced cases, irreversible parenchymal damage may lead to chronic respiratory impairment [47].

Chronic inflammation involving bronchi and bronchioles may result in airway wall thickening and bronchiectasis. Persistent lipid-laden macrophages and repeated inflammatory cycles contribute to the structural weakening of bronchial walls [48].

CT findings include cylindrical or varicose bronchiectasis within previously involved segments, often associated with peribronchovascular consolidation or residual ground-glass opacities. Air trapping may be present on expiratory images, reflecting small-airway involvement.

Airway remodelling may predispose patients to recurrent infections and chronic productive cough.

#### 3.7.2. Infectious and Acute Complications

Impairment of alveolar macrophage function due to lipid overload may reduce local immune defence mechanisms. Lipid-laden macrophages demonstrate altered phagocytic activity, potentially increasing susceptibility to opportunistic infections.

Superimposed bacterial, mycobacterial, or fungal infections have been described in association with chronic lipoid pneumonia. Radiologically, secondary infection may present as new consolidation within a previously stable lesion, cavitation, or increasing ground-glass opacity accompanied by systemic symptoms.

Distinguishing between progression of lipoid pneumonia and superinfection requires careful clinical correlation and, in some cases, microbiological evaluation [35].

Although uncommon, cavitation may develop in severe inflammatory cases or when superinfection occurs. The mechanism is thought to involve tissue necrosis secondary to intense inflammatory response or secondary infection.

On CT, cavitation appears as focal areas of central low attenuation within consolidation, occasionally with air-fluid levels. The presence of cavitation increases the complexity of differential diagnosis, as it may mimic necrotic malignancy or abscess formation [49].

In acute exogenous lipoid pneumonia, particularly following massive aspiration of hydrocarbon-based substances, alveolar rupture may occur due to intense inflammatory damage. This may result in the formation of pneumatoceles, pneumothorax, or pneumomediastinum [50].

These complications are typically identified on CT as air-containing cystic spaces or extrapulmonary air collections. While rare, their occurrence may indicate severe parenchymal injury and may be associated with worse clinical outcomes [46].

In extensive acute exogenous lipoid pneumonia, diffuse alveolar involvement may lead to hypoxemic respiratory failure. Imaging in such cases shows widespread consolidation and ground-glass opacities, occasionally resembling acute respiratory distress syndrome. Supportive management may require oxygen supplementation or mechanical ventilation [51].

#### 3.7.3. Pseudotumoral Evolution and Diagnostic Overtreatment

One of the most clinically significant complications is diagnostic misinterpretation as malignancy. Chronic lipoid pneumonia may evolve into mass-like lesions with irregular margins and heterogeneous enhancement. Inflammatory activity may produce increased uptake on fluorodeoxyglucose positron emission tomography, further raising suspicion for neoplastic disease.

This scenario may lead to repeated biopsies or even surgical resection when imaging findings are not recognized as potentially compatible with lipoid pneumonia. Histopathological confirmation often reveals lipid-laden macrophages rather than malignant cells [52].

Recognition of imaging features suggestive of intralesional fat and correlation with exposure history are therefore essential to prevent unnecessary invasive procedures.

### 3.8. Differential Diagnosis

The radiological diagnosis of lipoid pneumonia may be challenging due to its heterogeneous presentation and its ability to closely mimic a wide spectrum of infectious, inflammatory, and neoplastic lung diseases. Differential diagnosis should therefore be approached using a pattern-based strategy, integrating attenuation characteristics, lesion distribution, temporal evolution, and clinical context [53].

#### 3.8.1. Infectious Pneumonia

In both acute and chronic exogenous forms, lipoid pneumonia may closely resemble bacterial or viral pneumonia. Airspace consolidation, ground-glass opacities, and even crazy paving pattern may overlap significantly with infectious processes. However, infectious pneumonia typically lacks areas of negative attenuation within consolidation. Moreover, infectious opacities often demonstrate rapid radiologic evolution following antibiotic therapy, whereas lipid-related consolidations may persist despite clinical improvement [54].

In acute aspiration-related lipoid pneumonia, systemic inflammatory signs may further blur the distinction. Careful evaluation of attenuation values and detailed exposure history are critical in differentiating these entities [55].

#### 3.8.2. Organizing Pneumonia

Organizing pneumonia represents an important differential consideration, particularly in subacute or chronic presentations. Both conditions may present with peripheral or peribronchovascular consolidation and ground-glass opacities. However, organizing pneumonia typically demonstrates soft-tissue attenuation without intralesional fat density. The reverse halo sign may occasionally be present in organizing pneumonia but is not a characteristic feature of lipoid pneumonia. In ambiguous cases, the identification of negative Hounsfield unit values within consolidations strongly favours lipoid pneumonia [56].

#### 3.8.3. Pulmonary Alveolar Proteinosis

The crazy paving pattern, characterized by ground-glass attenuation with superimposed interlobular septal thickening, is a classic feature of pulmonary alveolar proteinosis and may also be observed in lipoid pneumonia. In alveolar proteinosis, however, attenuation values remain within soft-tissue range, and the distribution is often diffuse and symmetric. In contrast, lipoid pneumonia frequently demonstrates focal or lower lobe predominance and may reveal fat-density areas within consolidation [2]. Clinical context and bronchoalveolar lavage findings are decisive in differentiating these two entities.

#### 3.8.4. Primary Lung Carcinoma

Chronic lipoid pneumonia may evolve into focal mass-like lesions with irregular margins, spiculated contours, and heterogeneous enhancement. These features may strongly suggest primary lung malignancy, particularly adenocarcinoma. Furthermore, inflammatory activity may lead to increased fluorodeoxyglucose uptake on PET-CT, reinforcing suspicion of neoplasia.

The key distinguishing feature remains the presence of negative attenuation values within the lesion on non-contrast CT. However, in cases where inflammatory components elevate attenuation above the fat range, differentiation becomes more complex. MRI with fat-suppressed sequences may assist in confirming intralesional lipid content, although histopathological confirmation is often required [57].

Importantly, endogenous lipoid pneumonia may coexist with obstructing bronchogenic carcinoma. Therefore, the presence of lipid-laden macrophages does not exclude malignancy, and careful evaluation for an underlying obstructive lesion is mandatory [58].

#### 3.8.5. Pulmonary Hamartoma

Pulmonary hamartomas may also demonstrate fat attenuation on CT and therefore represent a relevant differential consideration. Unlike lipoid pneumonia, hamartomas typically present as well-circumscribed solitary nodules and may contain characteristic popcorn-like calcifications. They lack associated ground-glass opacities, crazy paving pattern, or surrounding inflammatory changes.

The morphological pattern and absence of inflammatory consolidation help distinguish hamartoma from lipid-related pneumonia [59].

#### 3.8.6. Metastatic Disease and Lipid-Containing Tumours

Certain metastatic lesions, particularly from liposarcoma or other fat-containing tumours, may demonstrate intralesional fat on imaging. However, metastatic lesions are usually multiple, well-defined nodules without associated airspace consolidation or inflammatory changes. Correlation with clinical history and the presence of systemic malignancy is essential in such scenarios [25].

### 3.9. Management and Radiological Follow-Up

The cornerstone of management in lipoid pneumonia is cessation of exposure to the offending lipid agent and, where applicable, mitigation of aspiration risk. In exogenous forms, identifying and eliminating the source—whether mineral oil, oil-based nasal drops, lipid-containing vaping products, or occupational exposures—is the primary therapeutic intervention and may lead to spontaneous radiological improvement or stabilisation in a substantial proportion of cases [47].

Systemic corticosteroids have been used in selected patients with progressive or symptomatic disease, based on the rationale of suppressing the foreign-body inflammatory reaction. However, evidence for their efficacy is limited to case reports and small case series, with no controlled trials available. The decision to initiate corticosteroids should therefore be individualised, weighing the potential benefit against the risk of immunosuppression in a patient already predisposed to aspiration [47].

Therapeutic bronchoalveolar lavage (BAL) has been proposed as an adjunctive treatment in cases with significant lipid burden, particularly in paediatric patients with mineral oil aspiration. A prospective study by Sias et al. demonstrated radiological improvement and restoration of BAL cellularity following multiple sequential lavages in children [27]. In a systematic review by Shang et al. encompassing 90 adults, therapeutic lung lavage—either segmental BAL or whole-lung lavage—resulted in clinical improvement in 96.7% of patients, with full radiological resolution in approximately 57% at last follow-up; procedural complications were rare but included transient and acute pulmonary oedema [50]. Whole-lung lavage is typically reserved for refractory cases with extensive bilateral disease.

Radiological follow-up with HRCT is an integral component of management. After exposure cessation, partial or complete resolution of consolidation may be expected over months to years, with the timeline varying according to the type and duration of lipid exposure, the degree of inflammation, and the extent of fibrotic remodelling. Follow-up imaging serves multiple purposes: (1) to document the expected trajectory of improvement or stabilisation, thereby reinforcing the diagnosis; (2) to detect progression or development of new focal features that would prompt re-evaluation and reconsideration of the differential diagnosis, including malignancy; and (3) to identify complications such as superimposed infection or progressive fibrosis. In general, a follow-up HRCT at 3–6 months after exposure cessation is reasonable in stable, asymptomatic patients. More frequent imaging is warranted in patients with worsening symptoms, new consolidation, or mass-like evolution. Where fibrotic sequelae are established, long-term surveillance may be appropriate to monitor respiratory function and parenchymal stability.

## 4. Conclusions

Lipoid pneumonia remains an uncommon and frequently underrecognized pulmonary condition with highly variable imaging manifestations. HRCT plays a central role in detection, particularly when intralesional fat attenuation can be demonstrated, while MRI may provide useful complementary information in selected equivocal or mass-like cases. A pattern-based radiological approach, integrated with clinical history and cytological or histopathological correlation, is essential to achieve an accurate diagnosis, avoid misinterpretation as infection or malignancy, and reduce unnecessary invasive procedures.

## Figures and Tables

**Table 1:** Reported prevalence of intralesional fat attenuation (or hypodense component) on CT or HRCT in lipoid pneumonia across representative series.

Study (Year), Design, Setting	Population	HRCT Criterion for Fat Attenuation	Frequency of Fat Attenuation or Hypodense Component
Samhouri et al. (2021), retrospective cohort, Chest [1]	Adults, *n* = 34	Qualitative “fatty attenuation” identifiable on chest CT	14/34 (41%)
Cozzi et al. (2021), retrospective HRCT series, Radiology: Cardiothoracic Imaging [29]	Adults, *n* = 10	Consolidation with adipose density, threshold < −40 HU	8/10 (80%)
Gondouin et al. (1996), multicentre retrospective series, ERJ [30]	Cases, *n* = 44	CT changes described as “hypodense” (fat measurement not always feasible, no HU threshold reported)	31/44 (71%) reported as “hypodense”
Zanetti et al. (2007), paediatric HRCT series, Pediatric Radiology [31]	Children, *n* = 17	Consolidations “usually with areas of fatty attenuation” (qualitative)	12/17 (70.6%) with fatty attenuation
Marchiori et al. (2007), adult HRCT series, Radiologia Brasileira [11]	Adults, *n* = 8	Consolidation with “fat densities,” measured range −34 to −74 HU	6/8 (75%)
Laurent et al. (1999), HRCT and MRI with pathologic correlation, European Radiology [32]	Adults, *n* = 7, 6 consolidations analysed	Consolidations classified as “fatty” versus non-specific low attenuation (qualitative)	3/6 lesions (50%) had “fatty” attenuation

**Table 2:** Differential diagnosis of lipoid pneumonia: Key imaging and clinical features. Provides a structured comparison of the key imaging and clinical features that distinguish lipoid pneumonia from its principal radiological mimics. The most reliable discriminator remains the presence of intralesional fat attenuation on non-contrast HRCT; however, in fat-negative cases, integration of exposure history, lesion distribution, temporal evolution, and BAL or histopathological data is essential.

Entity	CT Attenuation	Distribution	Associated Findings	BAL/Pathology	Key Distinguishing Feature
Lipoid pneumonia	−30 to −150 HU (fat) in 40–80%; may be +/soft tissue if fat-negative	Lower lobes, posterior; may be mass-like	Crazy paving, GGO, traction bronchiectasis; mass-like in chronic disease	Lipid-laden macrophages (foam cells); LLMI ≥ 100	Fat HU on non-contrast CT; exposure history (mineral oil, vaping); fat-sensitive MRI
Bacterial/viral pneumonia	Consolidation +20 to +50 HU; no fat attenuation	Lobar or segmental; acute onset	Air bronchograms, pleural effusion; fever, elevated CRP/PCT	Inflammatory cells; organisms on culture	Rapid evolution with antibiotics; no fat attenuation; absence of lipid exposure history
Organizing pneumonia	+20 to +50 HU; peribronchovascular or subpleural bands	Bilateral, peribronchovascular or subpleural; reversed halo sign	Reversed halo (atoll) sign; perilobular pattern	Intra-alveolar fibroblastic plugs (Masson bodies); no foam cells	Reversed halo sign; response to corticosteroids; no fat HU; no oil exposure
Pulmonary alveolar proteinosis (PAP)	+10 to +30 HU; no fat	Bilateral, geographic; crazy paving	Crazy paving pattern; normal lung volumes; no GGN	PAS-positive lipoproteinaceous material; no foam cells	Crazy paving without fat HU; milky BAL fluid; no exposure history; serum GM-CSF Ab
Primary lung carcinoma	Variable; soft tissue density; cavitation possible	Focal; mass or nodule; spiculated margins	Lymphadenopathy; pleural involvement; PET avid	Malignant cells on biopsy/EBUS	No fat HU; spiculated margins; PET hypermetabolism; no lipid exposure
Pulmonary hamartoma	Fat HU (similar to LP); calcification (popcorn)	Solitary, well-defined nodule; no consolidation	No surrounding GGO; no pleural effusion	Mixture of cartilage, fat, epithelium	Solitary nodule; popcorn calcification; no consolidation or exposure history
Liposarcoma metastases/fat-containing tumours	Fat HU, but multiple, nodular	Multiple bilateral nodules; well-defined	No consolidation; no GGO	Lipid-containing malignant cells; known primary	Multiple discrete nodules; known systemic malignancy; no aspiration history

Abbreviations: CT, computed tomography; HU, Hounsfield units; GGO, ground-glass opacity; BAL, bronchoalveolar lavage; LLMI, Lipid-laden Macrophage Index; PAS, periodic acid–Schiff; GM-CSF Ab, granulocyte-macrophage colony-stimulating factor antibody; EBUS, endobronchial ultrasound; PET, positron emission tomography. Note: fat attenuation in LP may be absent in fat-negative presentations (≈20–60% of cases) due to inflammatory admixture or fibrotic remodelling; clinical context and exposure history remain essential in all cases.

## Data Availability

The original contributions presented in this study are included in the article/Supplementary Materials. Further inquiries can be directed to the corresponding author.

## Supplementary Materials

The following supporting information can be downloaded at https://www.mdpi.com/article/10.3390/diagnostics16111693/s1. PRISMA 2020 Checklist.

## Abbreviations

The following abbreviations are used in this manuscript:

LP	Lipoid pneumonia
HRCT	High-resolution computed tomography
CT	Computed tomography
MRI	Magnetic resonance imaging
BAL	Bronchoalveolar lavage
HU	Hounsfield unit

## References

[1] Samhouri B.F., Tandon Y.K., Hartman T.E., Harada Y., Sekiguchi H., Yi E.S., Ryu J.H. (2021). Presenting Clinicoradiologic Features, Causes, and Clinical Course of Exogenous Lipoid Pneumonia in Adults. Chest.

[2] Sood N., Murin S. (2021). Lipoid Pneumonia: Fat Chance of Making the Diagnosis?. Chest.

[3] Banka R., Deshpande R.B., Udwadia Z.F. (2016). Lipoid Pneumonia After Prolonged Inhalation of Clarified Butter Made from the Milk of a Buffalo or Cow (Ghee). Indian J. Chest Dis. Allied Sci..

[4] Panse P.M., Feller F.F., Butt Y.M., Smith M.L., Larsen B.T., Tazelaar H.D., Harvin H.J., Gotway M.B. (2020). Radiologic and Pathologic Correlation in EVALI. Am. J. Roentgenol..

[5] Volk B.W., Nathanson L., Losner S., Slade W.R., Jacobi M. (1951). Incidence of Lipoid Pneumonia in a Survey of 389 Chronically Ill Patients. Am. J. Med..

[6] Butler S.G., Stuart A., Markley L., Feng X., Kritchevsky S.B. (2018). Aspiration as a Function of Age, Sex, Liquid Type, Bolus Volume, and Bolus Delivery Across the Healthy Adult Life Span. Ann. Otol. Rhinol. Laryngol..

[7] Beck L.R., Landsberg D. (2025). Lipoid Pneumonia.

[8] Morgan H.G. (1952). Endogenous Lipid Pneumonia. Edinb. Med. J..

[9] Byerley J.S., Hernandez M.L., Leigh M.W., Antoon J.W. (2016). Clinical Approach to Endogenous Lipoid Pneumonia. Clin. Respir. J..

[10] Laughlen G.F. (1925). Studies on Pneumonia Following Naso-Pharyngeal Injections of Oil. Am. J. Pathol..

[11] Marchiori E., Zanetti G., Nobre L.F., Takayassu T.C., Irion K.L. (2010). Lipoid Pneumonia Complicating Megaesophagus Secondary to Chagas Disease: High-Resolution Computed Tomography Findings. J. Thorac. Imaging.

[12] Hadda V., Khilnani G.C. (2010). Lipoid Pneumonia: An Overview. Expert Rev. Respir. Med..

[13] Hassan N., Battey T., Kurpiel B., Hanley M., Ropp A.M. (2025). Exogenous Lipoid Pneumonia Due to Aerosolized Essential Oils: An Unusual Source of Chronic Pneumonia Mimicking Neoplasm. Radiol. Cardiothorac. Imaging.

[14] El-Serag H.B., Sweet S., Winchester C.C., Dent J. (2014). Update on the Epidemiology of Gastro-Oesophageal Reflux Disease: A Systematic Review. Gut.

[15] Davidson K., Brancato A., Heetderks P., Mansour W., Matheis E., Nario M., Rajagopalan S., Underhill B., Wininger J., Fox D. (2019). Outbreak of Electronic-Cigarette-Associated Acute Lipoid Pneumonia—North Carolina, July–August 2019. MMWR Morb. Mortal. Wkly. Rep..

[16] Sangani R., Rojas E., Forte M., Zulfikar R., Prince N., Tasoglou A., Goldsmith T., Casuccio G., Boyd J., Olfert I.M. (2021). Electronic Cigarettes and Vaping-Associated Lung Injury (EVALI): A Rural Appalachian Experience. Hosp. Pract..

[17] Blount B.C., Karwowski M.P., Shields P.G., Morel-Espinosa M., Valentin-Blasini L., Gardner M., Braselton M., Brosius C.R., Caron K.T., Chambers D. (2020). Vitamin E Acetate in Bronchoalveolar-Lavage Fluid Associated with EVALI. N. Engl. J. Med..

[18] Chieng H.C., Ibrahim A., Chong W.H., Freed H., Fabian T., Saha B., Foulke L., Chopra A. (2022). Lipoid Pneumonia. Am. J. Med. Sci..

[19] Faggian G., Faggian R., Salzano M., Stavolo C., Di Nuzzo L., Carfora M., Coppola L., Cerrone M., Argenziano T., Argenziano A. (2026). Lipoid Pneumonia: A Narrative Review of Recent Developments. Ital. J. Med..

[20] Rebuli M.E., Rose J.J., Noël A., Croft D.P., Benowitz N.L., Cohen A.H., Goniewicz M.L., Larsen B.T., Leigh N., McGraw M.D. (2023). The E-Cigarette or Vaping Product Use-Associated Lung Injury Epidemic: Pathogenesis, Management, and Future Directions: An Official American Thoracic Society Workshop Report. Ann. Am. Thorac. Soc..

[21] Ding Y., Simpson P.M., Schellhase D.E., Tryka A.F., Ding L., Parham D.M. (2002). Limited Reliability of Lipid-Laden Macrophage Index Restricts Its Use as a Test for Pulmonary Aspiration: Comparison with a Simple Semiquantitative Assay. Pediatr. Dev. Pathol..

[22] Wang Y.X., Fang F., Guo Y.F., Li Y.M., Sun T.Y., Zhang M., Chen J., Fang B.M. (2017). [Analysis of 12 cases of exogenous lipoid pneumonia confirmed by pathology]. Zhonghua Jie He He Hu Xi Za Zhi.

[23] Papla B., Urbańczyk K., Gil T., Talar P., Kużdżał J. (2011). Exogenous Lipoid Pneumonia (Oil Granulomas of the Lung). Pol. J. Pathol..

[24] Guyard A., Cazes A. (2024). Pneumopathie lipidique exogène. Ann. Pathol..

[25] Liu Y., Wang M., Yu S.-H. (2026). Case Report and Comprehensive Literature Review: A Rare Instance of Lipoid Pneumonia. Medicine.

[26] Cox J.E., Choplin R.H., Chiles C. (1996). Case Report. Chemical-Shift MRI of Exogenous Lipoid Pneumonia. J. Comput. Assist. Tomogr..

[27] Fukushiro Y., Horimasu Y., Yamaguchi K., Sakamoto S., Masuda T., Nakashima T., Iwamoto H., Ohshimo S., Fujitaka K., Hattori N. (2025). Exogenous Lipoid Pneumonia Induced by the Excessive Use of Menthol-Containing Nasal Inhalers and Subsequent Acute Hypersensitivity Pneumonia Triggered by Domestic Environmental Antigens. Intern. Med..

[28] Sias S.M.A., Daltro P.A., Marchiori E., Ferreira A.S., Caetano R.L., Silva C.S., Müller N.L., Moreira J., Quirico-Santos T. (2009). Clinic and Radiological Improvement of Lipoid Pneumonia with Multiple Bronchoalveolar Lavages. Pediatr. Pulmonol..

[29] Cozzi D., Bindi A., Cavigli E., Grosso A.M., Luvarà S., Morelli N., Moroni C., Piperio R., Miele V., Bartolucci M. (2021). Exogenous Lipoid Pneumonia: When Radiologist Makes the Difference. Radiol. Med..

[30] Gondouin A., Manzoni P., Ranfaing E., Brun J., Cadranel J., Sadoun D., Cordier J.F., Depierre A., Dalphin J.C. (1996). Exogenous Lipid Pneumonia: A Retrospective Multicentre Study of 44 Cases in France. Eur. Respir. J..

[31] Zanetti G., Marchiori E., Gasparetto T.D., Escuissato D.L., Soares Souza A. (2007). Lipoid Pneumonia in Children Following Aspiration of Mineral Oil Used in the Treatment of Constipation: High-Resolution CT Findings in 17 Patients. Pediatr. Radiol..

[32] Laurent F., Philippe J.C., Vergier B., Granger-Veron B., Darpeix B., Vergeret J., Blanc P., Velly J.F. (1999). Exogenous Lipoid Pneumonia: HRCT, MR, and Pathologic Findings. Eur. Radiol..

[33] Rea G., Perna F., Calabrese G., Molino A., Valente T., Vatrella A. (2016). Exogenous Lipoid Pneumonia (ELP): When Radiologist Makes the Difference. Transl. Med. UniSa.

[34] Rea G., Lassandro F., Valente T. (2016). Neumonía lipoidea exógena en pacientes laringectomizados: ¿es el patrón de opacificación en vidrio esmerilado/en empedrado una reacción organizada de la neumonía capaz de predecir una mala evolución?. Arch. De Bronconeumol..

[35] Mohamed S., Bertolaccini L., Lombardi M., Di Tonno C., Sabalic A., Casiraghi M., Spaggiari L. (2025). Unmasking the Mimic: Lipoid Pneumonia Imitating Primary Lung Cancer—a Case Report Series of a Diagnostic Challenge. Front. Oncol..

[36] Marrocco A., Singh D., Christiani D.C., Demokritou P. (2022). E-Cigarette Vaping Associated Acute Lung Injury (EVALI): State of Science and Future Research Needs. Crit. Rev. Toxicol..

[37] Lee A.S., Ryu J.H. (2018). Aspiration Pneumonia and Related Syndromes. Mayo Clin. Proc..

[38] Betancourt S.L., Martinez-Jimenez S., Rossi S.E., Truong M.T., Carrillo J., Erasmus J.J. (2010). Lipoid Pneumonia: Spectrum of Clinical and Radiologic Manifestations. Am. J. Roentgenol..

[39] Chepuri R., Shah J., Sheinin Y., Kurman J. (2020). Exogenous lipoid pneumonia: When petroleum jelly does more than just moisturize. Chest.

[40] Antoon J.W., Hernandez M.L., Roehrs P.A., Noah T.L., Leigh M.W., Byerley J.S. (2016). Endogenous Lipoid Pneumonia Preceding Diagnosis of Pulmonary Alveolar Proteinosis. Clin. Respir. J..

[41] Goenka U., Jajodia S., Jash D., Ghosh S., Bandyopadhyay S. (2022). Acute Exogenous Lipoid Pneumonia: Unusual Presentation as Cavitating Lung Disease with Pneumothorax. Respir. Med. Case Rep..

[42] Barreto M.M., Rafful P.P., Rodrigues R.S., Zanetti G., Hochhegger B., Souza A.S., Guimarães M.D., Marchiori E. (2013). Correlation between Computed Tomographic and Magnetic Resonance Imaging Findings of Parenchymal Lung Diseases. Eur. J. Radiol..

[43] Molinari F., Bankier A.A., Eisenberg R.L. (2011). Fat-Containing Lesions in Adult Thoracic Imaging. Am. J. Roentgenol..

[44] Gibson G.J., Loddenkemper R., Lundbäck B., Sibille Y. (2013). Respiratory Health and Disease in Europe: The New European Lung White Book. Eur. Respir. J..

[45] Meyer K.C., Raghu G., Baughman R.P., Brown K.K., Costabel U., Du Bois R.M., Drent M., Haslam P.L., Kim D.S., Nagai S. (2012). An Official American Thoracic Society Clinical Practice Guideline: The Clinical Utility of Bronchoalveolar Lavage Cellular Analysis in Interstitial Lung Disease. Am. J. Respir. Crit. Care Med..

[46] Shang L., Gu X., Du S., Wang Y., Cao B., Wang C., for CAP-China Network (2021). The Efficacy and Safety of Therapeutic Lung Lavage for Exogenous Lipoid Pneumonia: A Systematic Review. Clin. Respir. J..

[47] Berghaus T.M., Haeckel T., Wagner T., Von Scheidt W., Schwaiblmair M.G. (2007). Endogenous Lipoid Pneumonia Associated with Primary Sclerosing Cholangitis. Lancet.

[48] Ayvazian L.F., Steward D.S., Merkel C.G., Frederick W.W. (1967). Diffuse Lipoid Pneumonitis Successfully Treated with Prednisone. Am. J. Med..

[49] Franzen D., Kohler M., Degrandi C., Kullak-Ublick G.A., Ceschi A. (2014). Fire Eater’s Lung: Retrospective Analysis of 123 Cases Reported to a National Poison Center. Respiration.

[50] Iwasaki Y., Sugihara R., Takagi O., Tsuya Y., Nakajima S. (1991). [A case of exogenous lipoid pneumonia showing a coin lesion with cavities]. Nihon Kyobu Shikkan Gakkai Zasshi.

[51] Yeung S.H.M., Rotin L.E., Singh K., Wu R., Stanbrook M.B. (2021). Exogenous Lipoid Pneumonia Associated with Oil-Based Oral and Nasal Products. Can. Med. Assoc. J..

[52] Brancaleone P., Delefortrie Q., Descamps O., Weynand B., Vanbever R., Detry G. (2023). Lipoid Pneumonia Associated with Polyethylene Glycol Chronic Aspiration. Am. J. Respir. Crit. Care Med..

[53] Mathai S.K., Rubinowitz A.N., Homer R.J., Detterbeck F., Herzog E.L. (2009). Of Lungs, Lipids, and Lollipops. Chest.

[54] Gurell M.N., Kottmann R.M., Xu H., Sime P.J. (2008). Exogenous Lipoid Pneumonia: An Unexpected Complication of Substance Abuse. Ann. Intern. Med..

[55] Spickard A. (1994). Exogenous Lipoid Pneumonia. Arch. Intern. Med..

[56] Marangu D., Gray D., Vanker A., Zampoli M. (2020). Exogenous Lipoid Pneumonia in Children: A Systematic Review. Paediatr. Respir. Rev..

[57] Khalil A., Majlath M., Gounant V., Hess A., Laissy J.P., Debray M.P. (2016). Contribution of Magnetic Resonance Imaging in Lung Cancer Imaging. Diagn. Interv. Imaging.

[58] Sundberg R.H., Kirschner K.E., Brown M.J. (1959). Evaluation of Lipoid Pneumonia. Dis. Chest.

[59] Krychniak-Soszka A., Lewandowska K., Skorupa W., Bartosiewicz M., Langfort R., Bestry I., Kuś J. (2005). [Exogenous lipid pneumonia--a report of four cases]. Pneumonol. Alergol. Pol..

