# Supplementary material for: Lipoid Pneumonia: HRCT and MRI Spectrum, Diagnostic Pitfalls, and Imaging-Based Diagnostic Workflow
Source: Diagnostics (Basel). 2026 May 30;16(11):1693. doi: 10.3390/diagnostics16111693 (PMC13256835; doi:10.3390/diagnostics16111693)
Supplement: Supplementary file 1 [file diagnostics-16-01693-s001.zip › diagnostics-4248487-supplementary.pdf]

PRISMA 2020 Checklist

Lipoid Pneumonia: HRCT and MRI Spectrum, Diagnostic Pitfalls, and Imaging-Based Diagnostic Workflow — A Systematic Review

Adorna M, Contino M, Libra A, Mauro LA, Castiglione DG, Mattina C, Mauceri C, Crimi C, Terminella A, Cusumano G, Gurrera A, Foti PV, Sambataro G, Basile A, Vancheri C, Palmucci S. Diagnostics 2026

Section / Topic	Item #	Checklist item	Location in manuscript (page / line)	N/A — justification
**TITLE**				
Title	*1*	*Identify the report as a systematic review.*	*p.1, line 2–3: 'A Systematic Review' added to title per editorial request. Original submission read 'A Pictorial Review'; revised per journal request to 'A Systematic Review'.*	
**ABSTRACT**				
Abstract	*2*	*See the PRISMA 2020 for Abstracts checklist.*	*p.1, lines 26–52. Structured abstract: Background/Objectives (lines 27–32), Methods (lines 33–39), Results (lines 40–47), Conclusions (lines 48–52).*	
**INTRODUCTION**				
Rationale	*3*	*Describe the rationale for the review in the context of existing knowledge.*	*p.2–3, lines 57–99. Rationale explicitly stated: no standardised imaging-based diagnostic pathway exists; CT fat attenuation variability (40–80%) not contextualised; emerging EVALI context. Knowledge gaps stated at lines 92–99.*	
Objectives	*4*	*Provide an explicit statement of the objective(s) or question(s) the review addresses.*	*p.2, lines 87–92: 'This review aims to provide a structured, imaging-centred analysis of LP, integrating pathophysiology, histopathology, and advanced imaging features, with particular emphasis on diagnostic pitfalls and complications.' Objectives: characterise HRCT/MRI spectrum; propose diagnostic workflow.*	
**METHODS**				
Eligibility criteria	*5*	*Specify the inclusion and exclusion criteria for the review and how studies were grouped for the syntheses.*	*p.3, lines 122–128. Inclusion: confirmed LP (histopathology / BAL Oil-Red-O / imaging+exposure*	

Section / Topic	Item #	Checklist item	Location in manuscript (page / line)	N/A — justification
			history); original CT/HRCT/MRI data; full-text peer-reviewed publication. Exclusion: no radiological data; abstract-only; animal/in vitro; autopsy-only diagnosis without ante-mortem imaging.	
Information sources	*6*	*Specify all databases, registers, websites, handsearching, organisations, reference lists, and other sources searched or consulted to identify studies. Specify the date when each source was last searched or consulted.*	*p.3, lines 101–118. PubMed/MEDLINE, Embase, Cochrane CENTRAL. Search period: January 1950 – 3 February 2025. Additional records via hand search of reference lists (line 117).*	
Search strategy	*7*	*Present the full search strategies for all databases and registers, including any filters and limits used.*	*p.3, lines 107–115. Full Boolean search string provided verbatim for PubMed/MEDLINE. Emtree+free-text for Embase; free-text for CENTRAL stated at lines 116–118. Complete search string also provided in Supplementary Table S1.*	
Selection process	*8*	*Specify the methods used to decide whether a study met the inclusion criteria of the review, including how many reviewers screened each record and data from each study, whether they worked independently, and if applicable, details of automation tools used in the process.*	*p.3, lines 120–122: 'Two authors independently screened titles and abstracts, with disagreements resolved by consensus.' Full-text assessment performed for all potentially eligible records.*	
Data collection process	9	Specify the methods used to collect data from reports, including how many reviewers collected data from each report, any processes for obtaining or confirming data from study investigators, and if applicable, details of automation tools used in the process.	p.3, lines 135–137: studies prioritised according to imaging quality, sample size, and availability of radiological–pathological or BAL correlation. Data synthesis performed narratively by the two senior authors. No automation tools used.	*Given the narrative design and predominance of case reports, formal data extraction forms were not used; key imaging data were extracted by consensus by the two lead authors.*
Data items	*10a*	*List and define all outcomes for which data were sought. Specify whether all results that were compatible with each outcome domain were sought (e.g. for all measures, time points, and analyses), and if not, the methods used to decide which results to collect.*	*Imaging outcomes: HRCT patterns (fat attenuation, consolidation, GGO, crazy-paving, nodules, fibrosis), MRI features (T1, fat-suppressed, chemical shift), and diagnostic accuracy data. Described throughout Section 3 (Discussion), pp.6–24.*	

Section / Topic	Item #	Checklist item	Location in manuscript (page / line)	N/A — justification
Data items	*10b*	*List and define all other variables for which data were sought (e.g. study characteristics, funding sources).*	*Study characteristics extracted: study design, population, imaging modality, LP type (exogenous/endogenous), diagnostic confirmation method, and key imaging finding. Summarised in Table 1 (fat attenuation prevalence data, p.10) and Table 2 (differential diagnosis features, pp.22–23).*	
Study risk of bias assessment	11	Specify the methods used to assess risk of bias in the included studies, including details of the tool(s) used, how many reviewers assessed each study, and whether the assessment was done independently.		*N/A — Given the narrative design and the predominance of case reports and small case series, formal risk-of-bias tools (e.g., RoB 2, ROBINS-I) were not applied. Study quality was assessed by consensus according to three criteria: imaging documentation quality, sample size, and availability of radiology–pathology or radiology–BAL correlation (p.3, lines 135–137).*
Effect measures	12	Specify for each outcome the effect measure(s) (e.g. risk ratio, mean difference) used in the synthesis or presentation of results.		*N/A — This is a narrative systematic review with no meta-analysis or pooled quantitative synthesis. Results are presented as proportions and descriptive frequencies (e.g., prevalence of fat attenuation, 41–80% across series; Table 1, p.10).*
Synthesis methods	*13a*	*Describe the processes used to decide which studies were eligible for each synthesis (e.g. tabulating the study characteristics and comparing against the pre-specified criteria).*	*All 59 included studies were considered for the narrative synthesis. Studies were grouped thematically by: (a) HRCT features; (b) MRI features; (c) diagnostic workflow; (d) complications; (e) differential diagnosis; (f) management. Grouping*	

Section / Topic	Item #	Checklist item	Location in manuscript (page / line)	N/A — justification
			described implicitly through the Discussion section structure (Sections 3.1–3.9).	
Synthesis methods	13b	Describe any methods required to prepare the data for presentation or synthesis, such as handling of missing data, data conversions, and data standardisation.		*N/A — No quantitative data preparation required. Fat attenuation frequencies are reported as published (Table 1, p.10). Methodological heterogeneity in HU thresholds across studies is explicitly acknowledged in Table 1 footnote and Section 3.5.1.*
Synthesis methods	*13c*	*Describe any methods used to tabulate or visually display results of individual studies and syntheses.*	*Tabular display: Table 1 (HRCT fat attenuation prevalence across 6 series, p.10); Table 2 (differential diagnosis comparative features, pp.22–23). Visual display: Figure 9 (diagnostic workflow, p.18); Supplementary Figure S1 (PRISMA flow diagram).*	
Synthesis methods	13d	Describe any methods used to synthesise results and provide a rationale for the choice(s). If meta-analysis was performed, describe the model(s), method(s) to identify the presence and extent of statistical heterogeneity, and software package(s) used.		*N/A — No meta-analysis performed. Narrative synthesis was chosen given the qualitative and descriptive nature of the available evidence (predominantly case reports and small case series). Imaging features are synthesised narratively following a pattern-based framework.*
Synthesis methods	13e	Describe any methods used to explore possible causes of heterogeneity among study results (e.g. subgroup analysis, meta-regression).		*N/A — No quantitative synthesis. Sources of heterogeneity in fat attenuation prevalence across studies are explored narratively in Section 3.5.1 (p.9–11) and in the Table 1 footnote (p.11), attributing*

Section / Topic	Item #	Checklist item	Location in manuscript (page / line)	N/A — justification
				variability to: differing HU thresholds, disease chronicity, inflammatory admixture, and selection bias in HRCT-focused series.
Synthesis methods	13f	Describe any sensitivity analyses conducted to examine robustness of the synthesised results.		*N/A — No quantitative synthesis performed; sensitivity analyses are not applicable to a narrative systematic review.*
Reporting bias assessment	14	Describe any methods used to assess risk of reporting bias arising from missing studies (e.g. funnel plot asymmetry) and risk of reporting bias within studies.		*N/A — Formal assessment of reporting bias (e.g., funnel plot, Egger test) is not applicable to a narrative synthesis without meta-analysis. Publication bias is acknowledged as a general limitation: positive or unusual cases are more likely to be published as case reports, and HRCT-focused series tend to over-represent fat-positive presentations (acknowledged in Section 3.5.1, p.10–11, and in Table 1 footnote).*
Certainty assessment	15	Describe any methods used to assess certainty (or confidence) in the body of evidence for an outcome.		*N/A — Formal certainty assessment (GRADE) was not performed, as stated explicitly in the Methods (p.3, lines 135–136). The predominantly case-report and small case-series nature of the evidence precludes formal grading. Evidence certainty is addressed qualitatively through the*

Section / Topic	Item #	Checklist item	Location in manuscript (page / line)	N/A — justification
				pattern-based framework and explicit identification of the limitations of each data source.
**RESULTS**				
Study selection	*16a*	*Describe the results of the search and selection process, including reasons for exclusion, using a flow diagram.*	*p.3–4, lines 129–134 (numerical results of screening process). Supplementary Figure S1 (PRISMA flow diagram): 196 records identified → 159 after deduplication → 57 full-text assessed → 59 studies included (40 database + 19 hand search).*	
Study selection	*16b*	*Cite studies that might appear to meet the inclusion criteria but which were excluded, and explain why they were excluded.*	*Excluded at full-text stage (n=17): studies without original imaging data (n=8, abstract-only publications n=5, language barrier n=4). Stated at p.3, lines 130–132.*	
Study characteristics	*17*	*Cite each included study and present its characteristics.*	*Included studies cited throughout the Discussion (refs 1–59). Key study characteristics (design, population, imaging modality, diagnostic confirmation, fat attenuation frequency) are presented in Table 1 (p.10) for the six primary fat attenuation series.*	
Risk of bias in studies	18	Present assessments of risk of bias for each included study.		*N/A — See Item 11. Formal risk-of-bias assessment was not performed given the narrative design. Study quality is discussed qualitatively; case reports are explicitly noted as carrying lower evidentiary weight than case series and cohort studies (p.4, line 137).*
Results of individual studies	*19*	*For all outcomes, present, for each study, (a) summary statistics for each group (if applicable) and (b) an effect estimate and its precision, usually with a confidence interval, for all the outcomes.*	*Quantitative data (fat attenuation frequencies) presented for individual series in Table 1 (p.10). Imaging features described for individual studies throughout Section 3.5 (pp.9–16). No*	

Section / Topic	Item #	Checklist item	Location in manuscript (page / line)	N/A — justification
			pooled effect estimates are presented (narrative synthesis).	
Results of syntheses	*20a*	*For each synthesis, briefly summarise the characteristics and risk of bias among contributing studies.*	*Synthesis of imaging features presented in Sections 3.5.1 (HRCT, pp.9–14) and 3.5.2 (MRI, pp.14–16). Study characteristics summarised in Table 1. Methodological limitations of contributing studies noted in Table 1 footnote and Section 3.5.1 (lines 324–334).*	
Results of syntheses	20b	Present results of all statistical syntheses conducted. If meta-analysis was done, present for each the summary estimate and its confidence interval, the amount of heterogeneity, and the I^2 statistic; if done, also report results of any investigation of possible causes of heterogeneity.		*N/A — No meta-analysis performed. Imaging feature frequencies are presented as individual study-level proportions in Table 1 (p.10). No pooled statistics computed.*
Results of syntheses	*20c*	*Present results of all investigations of possible causes of heterogeneity.*	*Sources of heterogeneity in fat attenuation prevalence discussed in Section 3.5.1 (pp.10–11) and Table 1 footnote (p.11): differing HU thresholds, disease chronicity, inflammatory admixture, and selection bias. Explicitly stated as a limitation of the tabulated evidence.*	
Results of syntheses	20d	Present results of all sensitivity analyses conducted.		*N/A — See Item 13f.*
Reporting biases	21	Present assessments of risk of reporting bias for an outcome.		*N/A — See Item 14. Publication bias is acknowledged narratively as a general limitation of the evidence base (Section 3.5.1, pp.10–11).*
Certainty of evidence	22	Present assessments of certainty (or confidence) in the body of evidence for an outcome.		*N/A — See Item 15. Certainty is addressed narratively throughout the Discussion. The evidence base is characterised as predominantly low-to-moderate certainty given the*

Section / Topic	Item #	Checklist item	Location in manuscript (page / line)	N/A — justification
				preponderance of case reports.
**DISCUSSION**				
Discussion	*23a*	*Provide a general interpretation of the results in the context of other evidence.*	*Section 3 (Discussion), pp.6–24. Interpretation provided for all major imaging features: fat attenuation (3.5.1), MRI (3.5.2), diagnostic workflow (3.6), complications (3.7), differential diagnosis (3.8), management (3.9). Contextualised against prior reviews (Hadda 2010, Betancourt 2010, Cozzi 2021, etc.).*	
Discussion	*23b*	*Discuss any limitations of the evidence included in the review.*	*Limitations of the evidence base discussed throughout: (a) fat attenuation variability contextualised in Section 3.5.1 and Table 1 footnote; (b) BAL limitations (LLMI sensitivity/specificity) discussed in Section 3.6 (pp.16–17); (c) evidence for corticosteroids noted as case-report level in Section 3.9 (p.23); (d) publication bias acknowledged.*	
Discussion	*23c*	*Discuss any limitations of the review processes used.*	*Limitations of the review methodology acknowledged: (a) narrative (not quantitative) synthesis; (b) absence of formal quality assessment; (c) case reports assigned lower evidentiary weight without formal grading; (d) single-language restriction applied only at post-screening stage. Stated in Methods, p.3, lines 135–137.*	
Discussion	*23d*	*Discuss implications of the results for practice, policy, and future research.*	*Conclusions (p.24, lines 737–743) and throughout Section 3.6 (diagnostic workflow, pp.16–18). Clinical implications: HRCT-first approach, targeted MRI use, BAL as adjunct, biopsy for refractory cases, follow-up imaging strategy. Research gap: absence of standardised LP*	

Section / Topic	Item #	Checklist item	Location in manuscript (page / line)	N/A — justification
			diagnostic criteria noted (lines 93–96).	
**OTHER INFORMATION**				
Registration and protocol	24a	Provide registration information for the review, including register name and registration number, or state that the review was not registered.		*Not registered. This systematic review was not pre-registered, as is common practice for narrative systematic reviews of imaging features without prospective protocol. Registration in PROSPERO was not performed given the retrospective, observational, and descriptive nature of the study.*
Registration and protocol	24b	Indicate where the protocol can be accessed, or state that a protocol was not prepared.		*No protocol was prepared or deposited prior to the search. The search strategy and eligibility criteria were agreed by the two lead authors (S.P. and M.A.) prior to the literature search, but were not formally registered.*
Registration and protocol	24c	Describe and explain any amendments to information provided at registration or in the protocol.		*N/A — No protocol was registered.*
Support	*25*	*Describe sources of financial or other support for the review, and the role of the funders or sponsors in the review.*	*p.24, lines 754–755: 'This research was partially funded by the Department of Medical Surgical Sciences and Advanced Technologies GF. Ingrassia, University of Catania.' No role of funder in study design, data collection, or manuscript preparation is declared.*	
Competing interests	*26*	*Provide a declaration of competing interests for the review authors.*	*p.24, line 760: 'The authors declare no conflicts of interest related to this manuscript.'*	
Availability of data, code,	*27*	*Report which of the following are publicly available and where they can be accessed: template data*	*No primary data were generated. Published data summarised in*	

Section / Topic	Item #	Checklist item	Location in manuscript (page / line)	N/A — justification
and other materials		*collection forms; data extracted from included studies; data used for all analyses; analytic code; any other materials used in the review.*	*Tables 1 and 2. Full Boolean search string provided in Methods Section 2.1 (p.3) and Supplementary Table S1. PRISMA flow diagram provided as Supplementary Figure S1. Institutional case imaging data cannot be publicly shared due to patient confidentiality, but de-identified demographic and diagnostic data are available upon reasonable request to the corresponding author.*	

Legend

N/A = Not applicable. Items not applicable to a narrative systematic review without meta-analysis are grayed out with explicit justification. The PRISMA 2020 statement (Page MJ et al., BMJ 2021;372:n71) permits narrative synthesis reviews to mark quantitative synthesis items as N/A with justification.

This checklist was compiled with reference to the submitted revised manuscript (version submitted for peer review, Diagnostics 2025). Page/line references correspond to the PDF version clear_manuscript_2902_pdf.pdf.
